# Female Heart Health: Is GPER the Missing Link?

**DOI:** 10.3389/fendo.2019.00919

**Published:** 2020-01-14

**Authors:** Leanne Groban, Quang-Kim Tran, Carlos M. Ferrario, Xuming Sun, Che Ping Cheng, Dalane W. Kitzman, Hao Wang, Sarah H. Lindsey

**Affiliations:** ^1^Department of Anesthesiology, Wake Forest School of Medicine, Winston Salem, NC, United States; ^2^Department of Internal Medicine-Molecular Medicine, Wake Forest School of Medicine, Winston Salem, NC, United States; ^3^Department of Physiology & Pharmacology, Des Moines University College of Osteopathic Medicine, Des Moines, IA, United States; ^4^Department of Surgery, Wake Forest School of Medicine, Winston Salem, NC, United States; ^5^Department of Physiology-Pharmacology, Wake Forest School of Medicine, Winston Salem, NC, United States; ^6^Department of Internal Medicine, Cardiovascular Medicine Section, Wake Forest School of Medicine, Winston Salem, NC, United States; ^7^Department of Pharmacology, Tulane University, New Orleans, LA, United States

**Keywords:** diastolic dysfunction, estrogen, heart failure with preserved ejection fraction, calcium homeostasis, chymase, inflammation, oxidative stress, LV remodeling

## Abstract

The G Protein-Coupled Estrogen Receptor (GPER) is a novel membrane-bound receptor that mediates non-genomic actions of the primary female sex hormone 17β-estradiol. Studies over the past two decades have elucidated the beneficial actions of this receptor in a number of cardiometabolic diseases. This review will focus specifically on the cardiac actions of GPER, since this receptor is expressed in cardiomyocytes as well as other cells within the heart and most likely contributes to estrogen-induced cardioprotection. Studies outlining the impact of GPER on diastolic function, mitochondrial function, left ventricular stiffness, calcium dynamics, cardiac inflammation, and aortic distensibility are discussed. In addition, recent data using genetic mouse models with global or cardiomyocyte-specific GPER gene deletion are highlighted. Since estrogen loss due to menopause in combination with chronological aging contributes to unique aspects of cardiac dysfunction in women, this receptor may provide novel therapeutic effects. While clinical studies are still required to fully understand the potential for pharmacological targeting of this receptor in postmenopausal women, this review will summarize the evidence gathered thus far on its likely beneficial effects.

## Introduction

Among measures of cardiac function, diastolic performance is one of the most comprehensive—integrating myocardial relaxation, mitochondrial bioenergetics, cardiomyocyte/myocardial structure, and left ventricular (LV) ejection with respect to proximal aortic distensibility—and is a potential barometer of cardiac health (1). LV diastolic function is impaired by all of the common pathological processes that affect LV function or produce LV hypertrophy or fibrosis, including hypertension, diabetes mellitus, obesity, sleep apnea, ischemia, aortic stenosis, and can occur before development of symptoms or changes in electrocardiogram and wall motion (2). The heart is designed to be a supple, elastic muscle that fills with blood easily at low pressure. Diastolic dysfunction with elevated filling pressures is a central feature of heart failure with preserved ejection fraction (HFpEF) (3) and disproportionally affects women with a sex ratio of about 2:1 (4–6). HFpEF is the most common form of heart failure (3, 7) and is outpacing other forms of heart failure as a result of the expanding elderly population (7–9).

Despite a marked female sex-specific predilection in HFpEF, relatively little is known regarding the mechanisms by which sex hormones, particularly the estrogens, and estrogen receptors (ERs) impact diastolic function. Over the last decade, we and others have explored the roles of the newest estrogen receptor, G protein-coupled estrogen receptor (GPER; previously known as GPR30), in the maintenance of cardiac function and structure after estrogen loss. In this review, the effects of pharmacologic activation of GPER by its specific agonist G1 on mitigating the adverse consequences of estrogen loss on relaxation, mitochondrial function, LV stiffness, and aortic distensibility will be presented. The influence of global and cardiomyocyte-specific GPER gene deletion on function and structure at the cardiomyocyte, whole heart, and conduit vessel levels will also be discussed.

## What is Diastolic Dysfunction?

**Figure 1:**
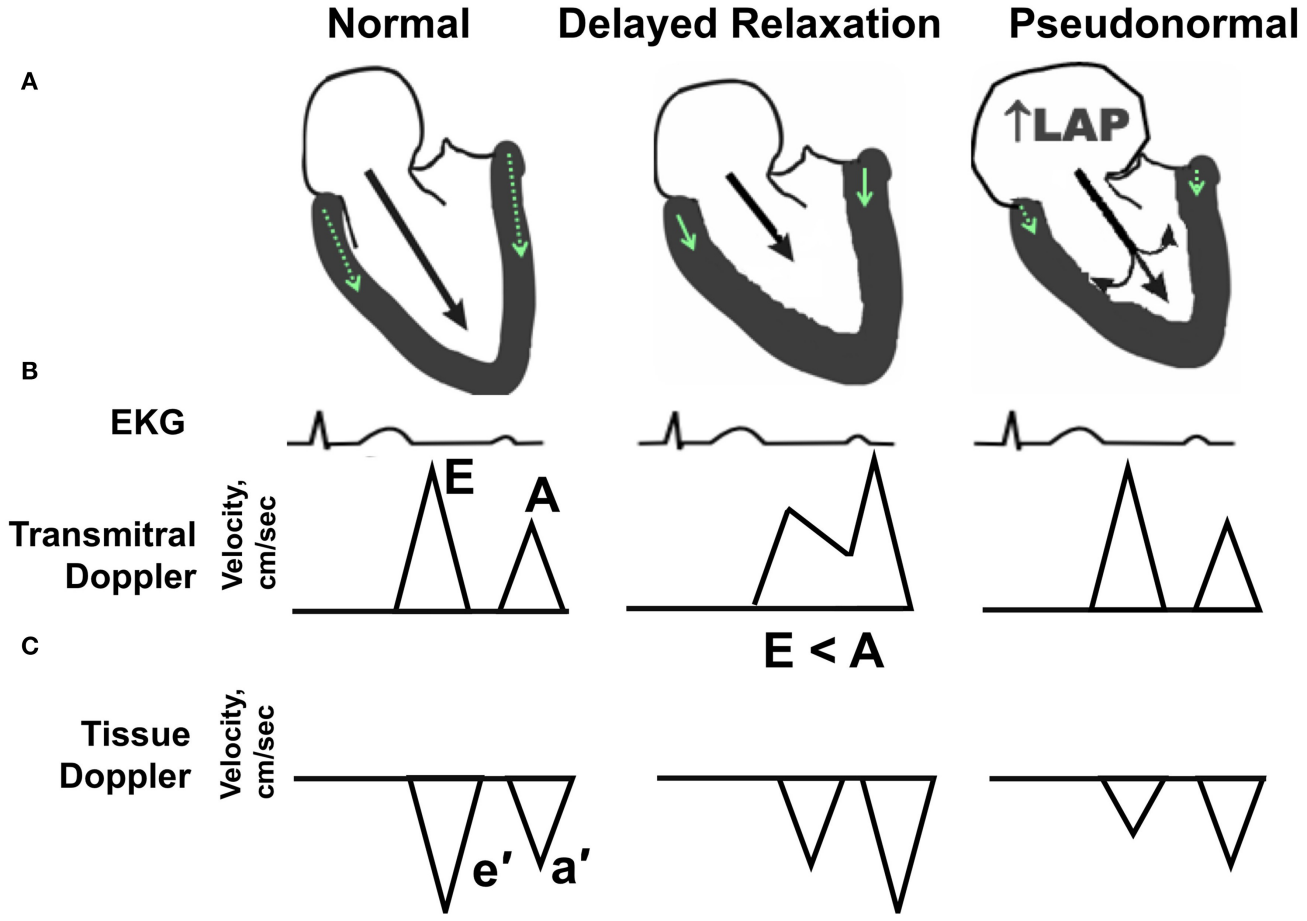
Echocardiographic hallmarks in the spectrum of diastolic dysfunction. **(A)** Schematic long-axis, sagittal view of the left atrium and left ventricle showing transmitral Doppler filling (black arrow) and septal and lateral mitral annular motion (small green arrow within LV wall) during early diastole. **(B)** Graphic representation of early and late transmitral Doppler-derived wave patterns in relation to the electrocardiogram (EKG). **(C)** Graphic representation of early and late tissue Doppler-derived mitral annular wave patterns. (Left) Normal diastolic function. The majority (80%) of left ventricular (LV) filling occurs during the early phase of diastole, as depicted by a relatively long black arrow extending from the mitral leaflets into the LV apex. The longer the arrow, the higher the relative velocity of early filling, or E wave, compared with late filling (A wave). Normally, E velocity is equal to or greater than A velocity. (Middle) Impaired relaxation or stage I diastolic dysfunction. With aging, mild hypertension or pressure overload, and/or ischemia, early filling (E wave) is impaired or reduced as depicted by a shorter extension of the black arrow into the LV apex. Also, late filling (A wave) is increased, due to a more vigorous atrial contraction to partly compensate and augment ventricular volume. The ratio of early-to-late-filling velocity is <1, or E < A, in this stage of diastolic dysfunction. Also, septal and lateral mitral annular velocities (e′) are reduced when compared with “normal” (green arrow). (Right) Pseudonormal pattern or Stage II diastolic dysfunction. With progressive worsening of diastolic dysfunction, LA size, and pressure increase. Because the LA functions as a reservoir to help maintain an appropriate atrioventricular pressure gradient during diastole, this increase in LA pressure (LAP) helps load blood into the non-compliant LV. With progressive worsening of diastolic dysfunction, LA size, and pressure increase. While the transmitral flow velocity profile appears normal, the mitral annular motion, or e′, remains reduced. In this situation, the mitral inflow profile is termed “pseudonormal”.

Diastolic dysfunction denotes a condition whereby the LV cannot fill adequately despite normal filling pressure. Slowing, delayed, and incomplete myocardial relaxation results from alterations in intracellular calcium handling, impairments in energy metabolism, and increases in LV stiffness due to hypertrophic and/or interstitial remodeling. Elevations in LV filling pressure initially compensate, but eventually pulmonary congestion develops as a result of increased left atrial (LA) pressure (10). While a wide range of diastolic function parameters can be obtained by Doppler-echocardiography (11), a simple composite of blood flow and tissue Doppler measures, as reviewed by ourselves and others (12–14) can sensitively detect and predict diastolic dysfunction in humans (15–17), non-human primates (18, 19), and preclinical rodent research models (20–25). The spectrum of diastolic dysfunction is portrayed schematically in Figure 1. Essentially, as described in detail by Nagueh and colleagues in the American Society of Echocardiography and European Association of Cardiovascular Imaging Guideline (11), the healthy adult LV fills primarily during the early filling phase of diastole, defined by transmitral Doppler E wave velocity, followed by a small contribution from atrial systole, defined by the late or transmitral A wave. Normally, E is equal to or greater than A. In addition, the longitudinal and radial myocardial fibers adjacent to the mitral annulus elongate and “twist” during early filling, creating a “suction-like” effect that helps propel blood into the LV. This motion of the mitral annulus during diastole is measured using tissue Doppler, and is termed e′. With increasing age (>50 years), in the initial stages of hypertension, and even in asymptomatic ischemia, early filling is slowed, delayed, or impaired and atrial contraction increases to partly compensate and augment ventricular volume. In this scenario, E wave velocity is less than the A wave velocity (e.g., E < A). In addition to the changes in filling dynamics, myocardial relaxation, assessed as e′, is reduced. With progressive worsening of diastolic dysfunction, LA size and pressure increase. Because the LA functions as a reservoir to help maintain an appropriate atrioventricular pressure gradient during diastole, this increase in LA pressure that occurs with progressive deterioration of diastolic function helps “load” blood into the non-compliant LV. In so doing, the transmitral flow velocity profile may appear normal (e.g., E > A); however, given that the mitral annular motion, or e′, remains reduced, the mitral inflow velocity profile represents a “pseudonormal” pattern, indicative of increased severity of diastolic dysfunction. Normally, the LV produces suction in order to fill while in the presence of advanced diastolic dysfunction, the left atrium produces loading in order to compensate and achieve adequate filling.

## What Evidence Supports a Role for Estrogen in the Maintenance of Diastolic Function?

The increased prevalence of HFpEF in older women compared with men of the same age appears related to the loss ovarian hormones, and primarily estrogens, that occur during menopause (7, 26). Epidemiologic evidence further suggests that premature or early natural menopause (27–29) and a shorter total reproductive duration positively associate with incident heart failure (30). Hall et al. (30) showed that the incidence of HFpEF was higher in postmenopausal women who were nulliparous, further suggesting a role of endogenous estrogens in the pathogenesis of the disease process. Importantly, diastolic dysfunction, the harbinger of HFpEF, was recently described as part of the “postmenopausal syndrome” (31). When compared with premenopausal women, postmenopausal women exhibit a higher prevalence of LV filling abnormalities. Moreover, when older women are compared with their age-matched male counterparts, the likelihood of manifesting more prominent diastolic dysfunction is increased (32–34). Findings from small clinical (35–39) and animal studies, as reviewed by us (40) and others (31, 41), document estrogen therapy efficacy in improving diastolic function and/or limiting increases in LV mass and interstitial remodeling after surgically induced or natural menopause. These data affirm estrogen's role in the preservation of diastolic function in the female heart.

## Estrogen Receptors in the Heart

Estrogen mediates its actions on the heart through three identified ERs. Estrogen receptor subtypes α (ERα) and β (ERβ) are classical nuclear hormone receptors, which bind estrogen and translocate to the nucleus to regulate target gene expression. However, molecular signaling is also induced by estrogen outside of the nucleus. While some intracellular signaling may be initiated by truncated forms of the steroid ERs (42), a membrane-bound ER distinct from ERα and ERβ was identified as the orphan receptor GPR30 before being renamed GPER (43, 44). GPER binds estradiol (E_2_) at a similar nanomolar affinity as ERα and ERβ and exerts comparable actions on calcium mobilization and phosphoinositide 3-kinase activation (45). ERα and GPER are expressed at similar levels in cardiac tissue from male and female rodents (46) as well as from humans (47). In contrast, reports of ERβ expression in the heart are conflicting, with ERβ mRNA detected in human cardiac tissue (48) but remaining below detectable levels in rodent cardiac tissue (46, 49). Based on observed improvement in cardiac function in response to E_2_ treatment in postmenopausal women (50) and in ovariectomized (OVX) rats (21), researchers have attempted to identify the primary receptor mediating estrogen's cardioprotective effects. Despite the inability to detect ERβ mRNA in some rodent models, administration of a novel ERβ agonist (βLGND2) attenuates angiotensin II-induced cardiac fibrosis (51) and genetic deletion of ERβ removes female sex-based cardioprotection in a model of pressure overload (52). These studies indicate that ERβ may be upregulated in the heart during disease or impact cardiac function through infiltrating cells rather than in cardiomyocytes. While studies using selective ERα and ERβ agonists indicate that both receptors induce cardioprotection (53), direct comparisons of genetic ERα and ERβ knockout (KO) mice indicate a dominant role for ERβ (54). However, assessing the double ER KO mouse in addition to each receptor KO individually showed no differences in infarct size, suggesting physiological redundancy or compensation (55).

GPER is expressed on the plasma and intracellular membranes of cardiac cells, including cardiomyocytes, cardiac fibroblasts, mast cells, and endothelial cells (56–59). To clarify the roles of GPER in the heart, pharmacologic approaches using the selective agonist G1 and antagonists G15 or G36 are commonly used. In Table 1, we summarize the ability of relevant hormones, natural estrogens, and drug molecules to bind to and activate signaling through GPER and ERα/β. G1 is the most commonly used tool for studying GPER. This non-steroidal, high-affinity (K_*d*_ = 11 nM) and highly selective GPER agonist was developed from a library of 10,000 molecules and does not activate the classical estrogen receptors at concentrations up to 10 μM (63). G15 and G36 are antagonists of GPER with low affinity binding to the classical estrogen receptors (64). While the exact signaling actions and transduction pathways of cardiac GPER are not completely understood, they are likely dependent on the cell type, site of action and the relative levels in comparison with the other estrogen receptors (46). The selective GPER agonist G1 modulates fast transduction pathways in the heart that are involved in (1) controlling intracellular calcium via actions on cardiac channels and pumps, (2) regulating phosphoinositide 3-kinase (PI3Ks) and extracellular signal-related kinases (ERKs), and (3) modulating cyclic adenosine monophosphate (cAMP) (see sections Effects of Estrogen and GPER Activation on I_Ca,L_ and Estrogen, GPER, and SERCA2a and Its Regulatory Proteins below). The rapid signaling events following GPER activation also lead to inhibition of the expression of cell cycle genes, such as cyclin B1 and CDK1, which are involved in cardiac fibroblast and mast cell proliferation and contribute to interstitial remodeling (see sections GPER Inhibits Interstitial Remodeling and GPER and Cardiac Chymase/Ang II below). Moreover, GPER activation by G1 reduces remodeling promoted by hypertrophic regulators, including angiotensin II and endothelin-1, via inhibition of 1/2 ERK signaling and upregulation of PI3K/Akt/mTOR pathways (see section GPER and Anti-hypertrophic Remodeling below).

**Table 1:** Ligands of GPER and ERs (60).

	**DPN**	**PPT**	**17β-Estradiol**	**Genistein BPA Nonylphenol DDT**	**Tamoxifen Raloxifene**	**ICI182,780**	**G-1**	**G15 G36**	**Quercetin (61, 62)**
ERα/β	++	++	++	++	+	–	n.d	n.d.	+
GPER	n.d.	+?	++	++	++	++	++	−−	++

*DPN, diaryl propionitrile; PPT, propylpyrazoletriol; BPA, bisphenol A; DDT, dichlorodiphenyltrichloroethane. Plus (+) symbol denotes weak agonist; Double plus (++) symbol denotes strong agonist; Minus (–) symbol denotes weak antagonist; Double minus (– –) symbol denotes strong antagonist; n.d., no reported data. Question mark (?) denotes interpret with care until confirmed by additional approaches*.

**Figure 2:**
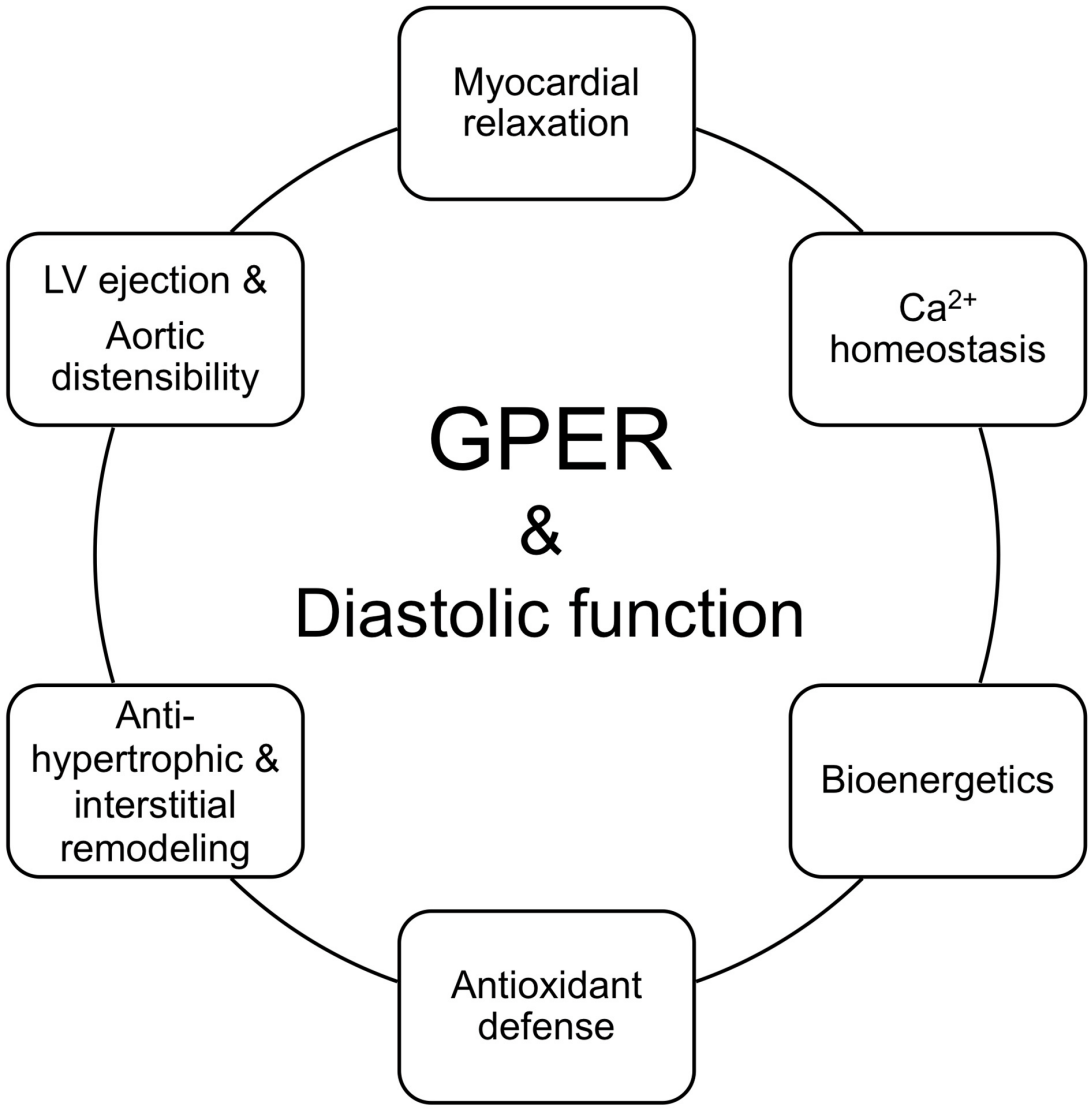
GPER in the functional circle of diastology of the female heart. Diastolic function is an appropriate barometer of overall heart health as it reflects cellular and subcellular events responsible for maintaining myocardial relaxation, cardiomyocyte calcium homeostasis, mitochondrial bioenergetics, antioxidant defense, left ventricular (LV) and myocyte structure, and proximal aortic distensibility. Loss of ovarian estrogens due to aging/menopause or surgery lead to impairments in myocardial/cardiomyocyte relaxation, increased mitochondrial reactive oxygen species and impaired oxidant defenses and bioenergetics, hypertrophic and/or interstitial remodeling, and aortic stiffening. Preclinical studies show that activation of the non-canonical estrogen receptor, GPER, by its agonist G1 or estradiol (E_2_) favorably regulates and likely integrates components of relaxation, mitochondrial function, LV structure, LV ejection, and aortic compliance, to preserve diastolic function in the female heart.

Since only a limited number of studies have explored the actions of GPER in diastolic function, future studies are needed to deepen our understanding of its effects in the various cardiac cell populations. Elucidation of these cell-type specific signaling mechanisms will help to clarify the therapeutic potential of cardiac GPER activation in preventing and/or halting the progression of cardiac diseases that involve diastolic dysfunction. Herein, where data exists, we include the signaling pathways of an activated GPER that are linked to the physiologic underpinnings of diastolic function preservation in the context of estrogen deprivation (Figure 2). Initial work showed that administration of the GPER agonist G1 prevents diastolic dysfunction and LV remodeling in OVX (25) and salt-loaded (65) mRen2.Lewis rats. To tease out more precise information about the functional role of GPER in the heart, we generated a novel conditional mouse model where GPER was specifically deleted in cardiomyocytes (23). Therefore, the remainder of the review will examine the evidence that links GPER to the preservation of myocardial relaxation and LV structure in the female heart after estrogen loss during hypertension, heart failure, and normal aging.

## GPER and Myocardial/Cardiomyocyte Relaxation

### Overview of Cardiac Ca^2+^ Machinery Involved in Contraction and Relaxation

Maintaining cardiomyocyte calcium concentration [Ca^2+^]_i_ within a tightly controlled range is critical for normal systolic and diastolic function. In adult cardiomyocytes, the L-type Ca^2+^ channel is the main pathway for Ca^2+^ influx. The Na/Ca^2+^ exchanger is quantitatively the most important pathway for Ca^2+^ efflux out of the cardiomyocyte. The sarcoplasmic/endoplasmic reticulum Ca^2+^ ATPase (SERCA) pumps and the sarcoplasmic reticulum (SR) Ca^2+^ release channels (ryanodine receptors) are pivotal in determining [Ca^2+^]_i_ and subsequent contraction and relaxation.

At the initiation of myocardial contraction, depolarization of the cardiomyocyte leads to activation of the inward Ca^2+^ current conducted via L-type Ca^2+^ channels Ca_v_1.2 (*I*_Ca,L_). This is the main trigger for Ca^2+^ release from the sarcoplasmic reticulum, termed Ca^2+^-induced Ca^2+^ release (CICR). CICR involves ryanodine receptors (RyR), of which RyR2 is the predominant myocardial isoform. *I*_Ca,L_ and CICR together increase the concentration of intracellular Ca^2+^ that binds to troponin C on myofilaments to initiate myocardial contraction.

Myocardial relaxation begins when ATP hydrolyzes and actin-myosin cross-bridges unlink. Removal of cytoplasmic Ca^2+^ and subsequent dissociation of Ca^2+^ from troponin is required for myocardial relaxation. This involves multiple components, including Ca^2+^ reuptake into the sarcoplasmic reticulum via SERCA2a (responsible for 70% cytoplasmic Ca^2+^ removal in humans) and activation of the sarcolemmal Na^+^/Ca^2+^ exchanger (NCX, 28%) and, to a lesser extent, plasma membrane Ca^2+^-ATPase (PMCA, 2%) (66). *I*_Ca,L_ also contributes to the filling status of the SR and myocardial relaxation. Other important processes allowing myocardial relaxation and diastolic ventricular filling include deactivation of the thin myofilaments, modulated by troponin and tropomyosin, and cross-bridge cycling, as recently reviewed (67).

We will briefly discuss the effects of estrogen on components of myocardial Ca^2+^ signaling, with a focus on mechanisms that regulate myocardial relaxation and the known role of GPER. A summary of the proteins/messengers examined in this context is provided in Table 2.

**Table 2:** Effect of E_2_ and GPER on proteins/messengers involved in Ca^2+^-dependent cardiac function.

**Ca^**2+**^ signaling protein involved**	**Assay**	**Model/Intervention/Treatment**	**Tissue/cells examined**	**Effects of intervention**
L-type Ca^2+^ channels (Ca_v_1.2)	Agonist-induced Ca^2+^ signal	G1 (68) G1 (69) G36 (69)	ISO-induced HFpEF myocytes (68) Male LV myocytes (69) Male LV myocytes (69)	Restored ISO-induced Ca^2+^ transient amplitude (68) ↓ ISO-induced Ca^2+^ signals (69) ↑ ISO-induced Ca^2+^ signals (69)
	Electrically stimulated Ca^2+^ transient	E_2_ (70–72) RVT (73) E_2_, raloxifene (71) E_2_/OVX (72) OVX (74)	Ventricular myocytes (70, 71, 73) ERα^−/−^, ERβ^−/−^ myocytes (71) LV apical myocytes (72) Ventricular myocytes (74)	↓ amplitude (70, 71, 73), delay recovery from inactivation (70) ↓ cell shortening (71) ↓ amplitude (71) ↑ amplitude (72) ↓ amplitude (74)
	ISO-induced cAMP Basal cAMP	E_2_ (75) OVX (72) OVX/E_2_ or G1 (72)	Perfused heart (75) LV apex (72) LV apex (72)	↓ cAMP (75) ↓ cAMP (72) Restored to sham level (72)
	Ca_v_1.2 mRNA immunoblotting	OVX (72) E_2_/OVX (72)	LV apical myocytes (72) LV apical myocytes (72)	↓ mRNA (72) Restored to sham level (72)
RyR2	Immunoblotting	G1/ OVX-HTN (25)	LV tissue (25)	No change (25)
	Caffeine-induced SR Ca^2+^ release	OVX (74)	Ventricular myocytes (74)	↑ SR Ca^2+^ release (74)
	^45^Ca^2+^ flux	OVX (76) E_2_/OVX (76) PKA (-)/ OVX (76)	LV myocytes (76) LV myocytes (76) LV myocytes (76)	↑^45^Ca^2+^ flux (76) Restored to sham level (76) Restored to sham level (76)
SR Ca^2+^-ATPase (SERCA)	Agonist-induced SR Ca^2+^ accumulation	G1 (24)	Saponin-skinned myocytes (24)	↑ Ca^2+^ accumulation (24)
	SR Ca^2+^ uptake	OVX (77) E_2_/OVX (77) Progesterone/OVX (77)	LV tissue (77) LV tissue (77) LV tissue (77)	↓ uptake (77) Restored uptake (77) Restored uptake (77)
	Immunoblotting	OVX (76) E_2/_OVX (76) G1/OVX old-aged (24)	LV myocytes (76) LV myocytes (76) LV tissue (24)	No change (76) No change (76) ↑ expression (24)
				
Phospholamban (PLB)	PLB mRNA PLB immunoblot	G1/OVX-HTN (25) OVX/MCT-PAH (20) OVX/G1/MCT-PAH (20)	LV tissue (25) RV tissue (20) RV tissue (20)	No change (25) ↓pPLB/PLB expression (20) Restored normal expression (20)
	Ser16 PLB phosphorylation	OVX (77)	LV tissue (77)	No change
	Thr17 PLB phosphorylation	OVX (77) E_2_/OVX (77) Progesterone/OVX (77)	LV tissue (77) LV tissue (77) LV tissue (77)	↓ Thr17 phosphorylation (77) Restored Thr17 phosphorylation (77) Restored Thr17 phosphorylation (77)
Na^+^/Ca^2+^ exchanger (NCX)	Na^+^-dependent Ca^2+^ uptake	OVX (76) E_2_/OVX (76) PKA(–)/OVX (76)	LV myocytes (76) LV myocytes (76) LV myocytes (76)	↑ Na^+^-dependent Ca^2+^ uptake (76) Restored to sham level (76) Restored to sham level (76)

*E2, estrogen; ER, estrogen receptor; HTN, hypertension; HFpEF, heart failure with preserved ejection fraction; ISO, isoproterenol; LV, left ventricular; MCT-PAH, monocrotaline-induced pulmonary arterial hypertension; OVX, ovariectomized; pPLB, phosphorylated phospholamban; PKA, protein kinase A; RV, right ventricular; RVT, right ventricular free wall thickness; RyR, ryanodine receptor; SR, sarcoplasmic reticulum*.

### Effects of Estrogen and GPER Activation on I_**Ca, L**_

While being a key contributor to systolic Ca^2+^ increase, *I*_Ca,L_ is also a main source for refilling SR Ca^2+^ in smooth muscle (78), and directly regulates diastolic Ca^2+^ level in ventricular myocytes (79). Thus, it significantly affects myocardial relaxation. Overall, there is abundant evidence that estrogen inhibits *I*_Ca,L_ via Ca_v_1.2 in cardiomyocytes.

The involvement of GPER in controlling myocardial contraction by mediating the inhibitory effects of E_2_ on *I*_Ca,L_ can now be deduced from observations made even before GPER was recognized as an ER. The negative inotropic effect of E_2_ was reported in the early 1990s. Indeed, E_2_ causes a decrease in cell shortening associated with reduced action potential duration; in patch-clamp and fluorescent measurements, E_2_ decreases the peak inward Ca^2+^ current and delays recovery of *I*_Ca,L_ from inactivation (70). The phytoestrogen resveratrol was later shown to inhibit the amplitude of electrically stimulated Ca^2+^ transients and cell shortening in ventricular cardiomyocytes (73). In line with earlier observations (70, 73), E_2_ (0.1–1 nM) reduces heart rate and pressure and cAMP production in the isolated perfused heart treated with isoproterenol; these effects are not inhibited by tamoxifen, an ERα/ERβ antagonist, and at the time were attributed to activation of an unknown membrane receptor (75). We now know that 4-OHT, a metabolite of tamoxifen, is a GPER agonist (45, 80). Later studies confirmed the inhibitory effect of E_2_ on *I*_Ca,L_ and further showed that genetic deletion of ERα or ERβ does not affect this inhibition (71). In the same studies, raloxifene, an antagonist of ERα/ERβ and agonist of GPER with a functional effective dose of 100 nM (44, 80), decreased *I*_Ca,L_ in cardiomyocytes from wild-type, ERα KO, and ERβ KO animals. Howlett's group also observed that electrically stimulated Ca^2+^ transients are larger in ventricular myocytes from OVX mice compared with sham female mice (74). We now know that a likely explanation for these observations was that they were mediated by GPER. Initial evidence supporting a role of GPER came from Tran's group, and indicated that GPER activation using G1 (0.001–1 μM) suppresses the isoproterenol-stimulated increases in LV contraction, Ca^2+^ signals, and *I*_Ca,L_ in intact hearts and in ventricular cardiomyocytes freshly isolated from male mice; these effects are associated with inhibition of protein kinase A (PKA)-dependent phosphorylation of Ca_v_1.2 (69). Taken together, the reported effects of E_2_ and GPER activation on cardiomyocyte *I*_Ca,L_ and Ca^2+^ transients have been consistently inhibitory and suggest that E_2_ and GPER prevent excessive cardiac contraction in response to acute stimuli.

In stressed cardiomyocytes, the picture appears to be different. The cardiac [Ca^2+^]_i_ regulatory systems are influenced by the activity of the sympathetic nervous system (SNS) via beta-adrenergic receptor (β-AR)-mediated, cAMP-dependent mechanisms. Estrogen alters gene expression of β-ARs and calcium-handling proteins (81). Preliminary data from Cheng's lab demonstrated that chronic *in vivo* G1 treatment restores normal myocyte basal and β-adrenergic receptor (β-AR)-mediated contraction, relaxation, and Ca^2+^ signals, leading to regression of LV dysfunction in a male mouse model of isoproterenol-induced HFpEF (68). These observations are consistent with reduced receptor sensitivity that is typically seen in heart failure. The ability of G1 to restore these parameters suggest that chronic GPER activation re-sensitizes cardiac β-AR regulation in this HFpEF model (68). These data also mirror those reported in a study of the apical myocardium, in which expression of the Ca_v_1.2α subunit and *I*_Ca,L_ were lower in apical myocytes from male or OVX mice compared with sham female mice, and E_2_ treatment of myocytes from OVX animals corrected these differences (72). The reduction in the amplitude of electrically stimulated Ca^2+^ transients in myocytes isolated from OVX female mice were restored by G1, to an extent similar to that achieved with E_2_ treatment. Moreover, blockade of GPER with G15 reversed the benefit of E_2_ while other ER antagonists had no effect. This data suggests that the protective effects of E_2_ on *I*_Ca,L_ are mediated in part through GPER in this model. Adrenergic stress-induced declines in contraction amplitude and calcium transients in OVX myocytes were also eliminated via E_2_/GPER as were decreases in cAMP concentration. Overall, existing data suggest that E_2_ and GPER activation reduce electrically stimulated or agonist-induced Ca^2+^ signals and contraction in the normal myocardium yet prevent the inhibition of these functions in stressed myocardium.

### Estrogen, GPER, and SERCA2a and Its Regulatory Proteins

SERCA2a is the main mechanism by which SR Ca^2+^ is refilled during diastole; it also is responsible for removing 70% of cytoplasmic Ca^2+^ in human cardiomyocytes (66). Several factors control SERCA2a activity. Phospholamban (PLB) is a trans-SR membrane protein that directly interacts with SERCA2a and reduces its activity by lowering its Ca^2+^ affinity (82). PLB phosphorylation at Ser16 and Thr17 in its cytoplasmic domain disinhibits SERCA2a. Phosphorylation of PLB at Ser16 is mediated by protein kinase A, while Thr17 is phosphorylated by Ca^2+^/CaM-dependent protein kinase II (CaMKII) (83, 84). By stimulating PLB phosphorylation, β-AR activation promotes SERCA activity, which increases the rate of Ca^2+^ sequestration in diastole and facilitates myocardial relaxation (83, 85). Sarcolipin (SLN) is another trans-SR membrane protein that regulates SERCA activity; genetic knockout of SLN enhances SR Ca^2+^ uptake and cardiac contractility (86). Similar to PLB, SLN reduces the Ca^2+^ affinity of SERCA2a, though the underlying mechanisms are still a source of debate.

GPER improves LV lusitropy in models of hypertension and aging (24, 25, 65). GPER may increase intracellular Ca^2+^ homeostasis and improve diastolic function by increasing either the expression and/or activity of SR Ca^2+^ regulatory proteins. Past reports showed that estrogen increases the expression of SERCA2a, while its expression is decreased in OVX animal models. We found no evidence for changes in SERCA2, PLB, calmodulin, or RYR2 gene or protein expression in cardiac tissues with chronic G1 treatment (50 μg/kg/day) of OVX mRen2.Lewis rats with systemic hypertension, despite improvements in myocardial relaxation (25). However, chronic activation of GPER with G1 (400 μg/kg/day) attenuates the adverse effects of monocrotaline (MCT)-induced pulmonary arterial hypertension (PAH) on SERCA2a and the ratio of phosphorylated PLB to total PLB in an OVX model (20). G1-mediated improvements in Ca^2+^ regulatory proteins are accompanied by a reversal in PAH-induced LV diastolic dysfunction, pulmonary artery flow, and right ventricular (RV) dysfunction when compared with vehicle-treated counterparts (20). In a normotensive aging model (26-month-old OVX-Brown Norway Fischer 344 rats), 8 weeks of G1 treatment reverses the adverse effects of age and E_2_ loss on myocardial relaxation, in part via increases in SERCA2 protein expression (24). In addition to changes in SERCA and PLB expression/phosphorylation profiles, the improvement in cardiac function with G1 treatment in these models could also be due in part to improvements in endothelial nitric oxide synthase (eNOS) activity and vascular tone. The effects of G1 on the vascular endothelium likely involve stimulation of Ca^2+^/calmodulin signaling network activities (87), including GPER activation *per se* (87, 88), upregulation of the Ca^2+^-dependent interaction between eNOS and calmodulin (87), improvement in the eNOS phosphorylation profile (87, 89), and optimization of vascular Ca^2+^ signaling via combined effects on influx (90) and efflux pathways (91).

To determine whether specific changes in SR Ca^2+^ uptake by GPER activation are associated with improvements in myocardial relaxation, we performed *ex vivo* studies in saponin-skinned muscle fascicles from 8-month-old female Wistar rats (24). SR Ca^2+^ content was evaluated by caffeine-induced tension under various loading conditions. Compared with vehicle, treatment with G1 increases SR Ca^2+^ accumulation in a concentration- and loading time-dependent manner suggesting that chronic GPER activation may increase cardiac Ca^2+^ mobilization not only by increasing the number of SERCA2 pumps, but by also augmenting SERCA activity (24). These data are consistent with a report on an OVX rat model; in that study, 10 weeks after OVX, SR Ca^2+^ uptake is reduced, with decreased SERCA activity and expression level, and Thr17 phosphorylation of PLB is reduced but Ser16 phosphorylation was unchanged. Interestingly, supplementation with either E_2_ or progesterone prevents the OVX-related reductions in cardiac SERCA expression and activity and Thr17 PLB phosphorylation (77). However, Yang et al. (92) reported that cardiomyocytes from OVX guinea pigs have 22% larger SR Ca^2+^ stores and higher frequency of Ca^2+^ sparks and waves than sham animals; addition of E_2_ prevents these changes. Similarly, Howlett's group showed that the caffeine-induced SR Ca^2+^ release signal, an indirect indicator of SR Ca^2+^ content, is larger in cardiomyocytes from OVX than from sham C57BL/6 mice (74). While these results appear to be contradictory, it is important to note that, in addition to differences in model species, treatment conditions, and dosing, the loss of progesterone with OVX may also play a significant role in the observed effects in each study, as suggested by data from Bupha-Intr and Wattanapermpool (77). Overall, studies using the specific GPER agonist G1 support a role for GPER in influencing SR Ca^2+^ uptake in the heart to improve diastolic function. Further studies using GPER KO models, especially cardiac-specific GPER^−/−^ cardiomyocytes, will provide further insights.

### Estrogen and the Na^+^/Ca^2+^ Exchanger

The NCX is responsible for removal of ~28% of cytoplasmic Ca^2+^ in human cardiomyocytes and as such is an important determinant of diastolic function. However, the effects of estrogen on cardiac NCX are unclear. Estrogen has been reported to increase, have no effect, or decrease NCX expression. In one study, NCX expression was increased by E_2_ and was decreased in untreated OVX rats (93). In other studies, no change was observed in the expression of NCX expression by estrogen treatment or OVX (25, 94). Kravtsov et al. (76) determined NCX activity in the heart from OVX rats. In their study, NCX activity, measured as Na^+^-dependent ^45^Ca^2+^ uptake, was increased by OVX, and E_2_ replenishment abolished this increase (76). Mechanistically, the effect of E_2_ loss and restoration was associated with changes in PKA-mediated activation of NCX and not on changes in the expression level of NCX. However, no study to date has specifically examined if GPER activation mediates the effects of E_2_ on NCX activity in cardiac tissue.

## GPER, Bioenergetics, and Mitochondrial ROS

Diastolic function is an energy-requiring process in that ATP is necessary for the sequestration of cytoplasmic Ca^2+^ back into the SR during diastole. As the heart possesses the highest content of mitochondria of any tissue (95), even slight alterations in mitochondrial cellular energy production contribute to impairment of myocardial relaxation. Indeed, an increasing body of literature suggests that abnormalities in cardiomyocyte mitochondrial function and structure are important factors in the pathogenesis of HFpEF (96, 97). Specifically, elevated and pathologic reactive oxygen species (ROS) production has been implicated in mitochondrial damage, resulting in a mismatch between ATP production and energy demand, while also activating signaling pathways that further contribute to LV remodeling, all of which lead to diastolic dysfunction.

The role of mitochondrial dysfunction in ROS accumulation and/or alterations in mitochondrial bioenergetics in the estrogen-deficient heart is still emerging, as are the mitochondria-related effects of E_2_ that are mediated through GPER (98). In a recent general population study (Flemish Study on Environment, Genes, and Health Outcomes), individuals with normal and abnormal diastolic function were found to have different levels of circulating metabolites indicative of energy substrate utilization and protection again oxidative stress (99). In another cohort from that study (100), mitochondrial DNA (mtDNA), a circulating marker of mitochondrial dysfunction, was positively associated with female sex, while mtDNA levels were reduced in women receiving estrogen/progesterone treatment. With regard to cardiac pathology, one preclinical study using OVX CTnT-Q92 transgenic mice as a model of human hypertrophic cardiomyopathy showed that E_2_ replacement reduces oxidative damage and improved decrements in mitochondrial bioenergetics and diastolic function (101). Potential mechanisms underlying the estrogen-mediated protection from mitochondrial-derived oxidative injury include decreasing ROS accumulation by increasing respiratory chain efficiency; reducing apoptotic leakage of cytochrome C; upregulating mitochondrial antioxidant enzymes such as manganese superoxide dismutase (MnSOD), catalase, and glutathione peroxidase; and decreasing NADPH oxidases (NOX4) (102–105). Pharmacologic interventions in isolated cardiomyocytes and in ischemia-reperfusion-challenged hearts indicate that GPER activation by G1 reduces oxidative stress by limiting cytochrome C release and inhibiting mitochondrial pore opening, respectively (106–108). Our data from the novel cardiomyocyte-specific GPER KO female mouse developed in the Groban laboratory extend these findings with more direct evidence of the potential importance of cardiac GPER in the maintenance of mitochondrial processes that counteract ROS accumulation in the female heart (23). In brief, treatment of female GPER KO mice with the mitochondrial antioxidant MitoQ attenuates the adverse effects of cardiomyocyte GPER deletion on myocardial relaxation, filling pressures, interstitial remodeling, and oxidative damage (109). MitoQ also limits the genomic responses to increased oxidative stress and decreases oxidant defense related to cardiomyocyte-specific GPER deficiency (109). Taken together, GPER appears to have a regulatory role in aspects of mitochondrial function that balance ROS formation and antioxidant defense, which in turn has the potential to impact intracellular calcium homeostasis (110), thereby contributing to the maintenance of diastolic function after estrogen loss.

As diastolic dysfunction accounts for half of what drives HFpEF symptoms and adverse clinical events, it is also important to consider the impact of systemic inflammation, coronary microcirculatory disturbances, skeletal muscle weakness, pulmonary disease, and renal dysfunction (111). Indeed, the most current paradigm of HFpEF goes well beyond diastolic dysfunction. For instance, systemic inflammation, oxidative stress, and/or endothelial dysfunction contribute to capillary rarefraction and mitochondrial dysfunction in skeletal muscle and myocardium of HFpEF patients (112–114), impairing oxygen delivery and utilization, and adversely affecting exercise tolerance (115, 116). While it is not entirely clear what role estrogen deficiency and/or GPER deactivation might have on these extracardiac parameters linked to HFpEF (117), future preclinical studies focusing on this paradigm may reveal therapeutic strategies that can be personalized to prevent the development of this disorder in postmenopausal women.

## GPER and LV Structure

In addition to the cellular mechanics of myocardial relaxation and subcellular mitochondrial energy-producing processes required, the structure of the myocardium at both cardiac muscle cell and LV chamber levels determines LV ventricular distensibility and stiffness. At the cellular level, isolated cardiomyocytes from preclinical models of diastolic heart failure exhibit increases in diameter without changes in length, which correspond to increases in LV wall thickness with normal or near-normal end diastolic volumes (118), a pattern indicative of concentric LV remodeling. *In vitro* cardiomyocyte functional data from patients with HFpEF further indicate increased stiffness and decreased distensibility, with resting tensions two times that of normal cardiomyocytes (119, 120). Translating this to the tissue level, a relatively stiff, non-distensible ventricle requires higher pressures to achieve filling of a given volume. Conventional and tissue Doppler echocardiographic techniques estimate filling pressure (see section What is Diastolic Dysfunction? above and Figure 1). The effects of GPER activation on components of LV remodeling, and potential mechanisms in the context of the renin angiotensin system and local inflammatory/immune processes, are presented in Table 3.

**Table 3:** Anti-remodeling effects of GPER activation in cardiac tissue and cells.

**Species or cells**	**Models/Strains**	**G1 treatment**	**Effects of intervention**
Rat	mRen2.Lewis rats/OVX (25)	s.c., 50 μg/kg/day, for 2 weeks	Limited OVX-induced ↑ LV filling pressure, LV mass, wall thickness, interstitial collagen deposition, and cardiac ANF and BNP mRNA levels
	mRen2.Lewis rats/high salt diet (65)	s.c., 40 nmol/kg/hr, for 2 weeks	Improved myocardial relaxation and reduced cardiac myocyte hypertrophy and wall thickness
	F344BN old-aged rats/OVX (24)	s.c. 100 μg/kg/day, for 8 weeks	Reversed adverse effects of age and estrogen loss on myocardial relaxation and interstitial collagen deposition
	Wistar rats/OVX + monocrotaline-induced pulmonary hypertension (20)	s.c., 400 μg/kg/day, for 14 days after the onset of disease	Limited adverse effects of pulmonary hypertension on RV interstitial fibrosis, RV free wall thickening, and LV diastolic function
	Wistar rats/myocardial infarction (121)	50 μg/kg per day, gastric gavage, for 4 weeks	Attenuated LV hypertrophy, assessed by cardiomyocyte size, to an extent similar to E_2_
	Wistar rats/OVX and diabetes mellitus (122)	i.p. injection, 50 μg/kg, every 4 days for 4 weeks	Improved cardiac weight, atherogenic and cardiovascular risk indices; meanwhile GPER antagonism with G15 exacerbated cardiac weight and atherogenic indices
	SD rats/OVX + ISO-induced heart failure (123)	s.c.,120 μg/kg·d, for 14 days	Decreased cardiac BNP, reduced cardiac fibrosis, and enhanced contraction
Mouse	C57BL/6 mice/OVX & myocardial infarction (124)	i.p. injection, 35 μg/kg/d, for 4 weeks	Reduced myocardial fibrosis and infarct area
	Ramp3^+/+^ and Ramp3^−/−^ (125)	s.c., 0.1 mg/kg/day, for 40 days	Reduced perivascular fibrosis and cardiomyocyte area in RenTgMK/Ramp3^+/+^ male mice
Neonatal rat cardiomyocytes	ET-1 (100 nmol/l) for 48 h (126)	10 nmol/l for 48 h	Abolished hypertrophic actions of ET-1, which was reversed by G15; siRNA silencing of GPER inhibited antihypertrophic effect of E_2_
	100 nM of Ang II for 24 h (127)	1,000 nM for 24 h	Attenuated Ang II-induced cardiomyocyte hypertrophy and downregulated mRNA levels of ANF and BNP
H9c2 cardiomyocytes	Ang II (10^−7^M) for 24 h (25)	10^−7^ M for 24 h	Inhibited Ang II-induced hypertrophy, evidenced by reductions in cell size, protein content per cell, and ANF mRNA levels; G15 inhibited protective effects of G1 or E_2_
Adult rat cardiac fibroblasts	Growth medium with 10% FBS (57)	0.01–10 μM for 24 h	Inhibited proliferation of rat cardiac fibroblasts

*ANF, atrial natriuretic factor; Ang II, angiotensin II; BNP, brain natriuretic peptide; E_2_, estradiol; ET-1, endothelin-1; FBS, fetal bovine serum; i.p., intraperitoneal; ISO, isoproterenol; LV, left ventricular; OVX, ovariectomized; RV, right ventricular; s.c., subcutaneous; μg, micrograms; SD, Sprague Dawley*.

### GPER and Anti-hypertrophic Remodeling

Preclinical studies reveal that GPER activation by E_2_ or G1 prevents hypertrophic remodeling, independent of its effects on blood pressure. We have shown that high salt or estrogen deprivation in hypertensive mRen2.Lewis rats increases LV mass, wall thickness, and myocyte size and is attenuated by chronic G1 treatment (25, 65). Moreover, ventricular hypertrophy assessed by cardiomyocyte size after infarction by coronary ligation in OVX Wistar rats is attenuated to a similar extent by G1 and E_2_ (121). Lee et al. (121) further showed that GPER and ERα activation converge to elicit post-ischemic antihypertrophic remodeling via a PI3K/Akt/eNOS-dependent pathway. In cultured primary neonatal cardiomyocytes (127) and H9c2 cells (25), GPER activation by G1 attenuates angiotensin II (Ang II)- and endothelin-1 (ET-1)-induced hypertrophy, respectively, as demonstrated by reductions in atrial natriuretic factor (ANF) and brain natriuretic peptide (BNP) mRNA expression levels, cell size, and protein content. The protective effects of GPER involved inhibition of ERK1/2 signaling and an upregulation of the PI3K/Akt/mTOR pathway (127). The latter is known to effect autophagy, which is important in the preservation of cell homeostasis. In another study using neonatal cardiomyocytes, ET-1–induced hypertrophy was prevented by E_2_/GPER via inhibition of ERK1/2 signaling (126).

### GPER Inhibits Interstitial Remodeling

Alterations in the extracellular matrix, specifically increases in collagen, with corresponding increments in the width and continuity of fibrillar components (128), further contribute to diastolic dysfunction through increases in chamber stiffness. Interstitial and perivascular collagen deposition are enhanced in physiopathologic situations that commonly manifest diastolic dysfunction such as aging, hypertension, pressure overload hypertrophy, and estrogen loss. E_2_ or GPER activation with G1 prevents increases in OVX-related effects on profibrotic gene expression, fibroblast proliferation, and collagen deposition in rodent and non-human primate models of normative cardiac aging, hypertension, and pulmonary hypertension (19–21, 24). However, it is worth mentioning that increased cardiac collagen after estrogen loss may not be universal, nor is its effect on increasing passive chamber stiffness (129). Whether length of time of estrogen deprivation, animal species and strain, and the type physiologic stress account for these discrepancies is not entirely clear. Nonetheless, *in vitro* studies demonstrate the capacity of E_2_ to regulate the proliferation of cardiac fibroblasts and their collagen production (130), effects that are deemed to be partly mediated by GPER. We recently confirmed GPER expression in cardiac fibroblasts of male Sprague Dawley rats and further demonstrated the efficacy of G1 on inhibiting cardiac fibroblast proliferation in a dose-dependent manner (57). These findings were confirmed *in vivo* in OVX-mRen2.Lewis females, in which 2 weeks of G1 treatment limits estrogen deficiency-induced increases in LV cardiac fibroblast number, proliferation, and gene expression levels of the cell cycle proteins, CDK1, and Cyclin B1 (57).

### GPER and Cardiac Chymase/Ang II

Activation of the renin angiotensin system (RAS) is one mechanism for LV hypertrophic and interstitial remodeling that contributes to LV stiffness and diastolic dysfunction. Indeed, Ang II is involved in tissue remodeling and the induction of fibrosis (131). While RAS blockade is a widely used approach to treat heart failure, including HFpEF (132–134), the clinical benefits gained from RAS blockers in halting or reversing disease progression has fallen short of expectations (135–138). These drugs may have limited ability to suppress Ang II synthesis at the intracellular spaces where Ang II is formed and exerts its trophic and profibrotic actions (139, 140). Findings from the Ferrario lab (140–144) and others (145–148) suggest that chymase, not angiotensin-converting enzyme (ACE), is the major Ang II-forming enzyme in both human and rat hearts, and produces Ang II from the substrate angiotensin I (Ang I) or angiotensin-(1–12) [Ang-(1–12)].

With respect to estrogen status (intact vs. OVX) and LV diastolic dysfunction, we demonstrated a positive relationship between cardiac chymase-forming Ang II and echo-derived filling pressures in normotensive Wistar Kyoto female rats (149) and hypertensive mRen.Lewis rats (150). OVX-related increases in chymase and Ang II expression were further associated with increases in cardiac fibrosis. Because mast cells are a major source of chymase (151, 152) and generate Ang II from Ang I or Ang-(1–12) (153), we also determined the impact of mast cell inhibition by the mast stabilizer cromolyn sulfate on OVX-induced diastolic dysfunction (154). In brief, 8 weeks of cromolyn sulfate administered subcutaneously to OVX-BNF344 rats attenuates the adverse effects of estrogen loss on diastolic function, interstitial collagen deposition, and collagen type 1A mRNA levels. Even though cardiac chymase activity in OVX rats is not overtly reduced by cromolyn (*P* < 0.06), cardiac Ang II content is reduced when compared with OVX vehicle, suggesting a role for mast cell derived-factors and chymase/Ang II in the progression of cardiac aging and diastolic dysfunction after estrogen loss (154). Indeed, E_2_ treatments favorably regulate cardiac mast cell number and prevent the adverse effects of OVX on cardiac remodeling and LV function in an Ang II-dependent rodent model of hypertension and LV diastolic dysfunction (150) and in models of surgically induced pressure overload (155) and volume overload (156, 157).

Although the mechanisms by which estrogen regulates cardiac mast cell number are not entirely clear, it appears to be mediated in part through GPER. Findings from us suggest that GPER is expressed in RBL-2H3 mast cells (58) and that GPER activation by its agonist G1 inhibits serum-induced proliferation of these cells through interaction with the cell cycle protein CDK1. GPER blockade by G15, but not by ERα or ERβ antagonists, completely prevents E_2_-induced inhibition of mast cell proliferation (58). This effect was confirmed *in vivo* in OVX-mRen2.Lewis rats; 2 weeks of G1 treatment decreases cardiac mast cell number and chymase expression/Ang II levels, and limits gene and protein expression of cell cycle proteins (58). Taken together, these data suggest that the inhibitory effects of GPER on extracellular matrix remodeling may in part involve cardiac mast cell chymase/Ang II modulation.

### GPER and Cardiac Inflammation

Another indirect way by which GPER activation could prevent OVX-induced remodeling and diastolic dysfunction is through modulation of local inflammatory defense mechanisms (111, 158).

Using cardiomyocyte-specific GPER KO mice (23), we found an intriguing relationship between loss of cardiac GPER and the NLRP3 inflammasome, which includes NLRP3, caspase-1, interleukin-1β (IL-1β), and IL-18. The NRLP3 inflammasome is formed and activated by various stimuli, including oxidative stress, and participates in the pathogenesis of hypertension, diabetes, atherosclerosis, myocardial infarction, heart failure, and other cardiovascular diseases (159). Characterization of innate immunity gene transcripts in hearts from 6-month-old cardiomyocyte-specific GPER KO mice and their GPER-intact wild-type littermates revealed that expression of NLRP3 and IL-18 are increased nearly three-fold (22). The importance of NLRP3 upregulation in GPER KO-induced heart failure was further confirmed in an *in vivo* study showing that, compared with vehicle-treated KO mice, 8 weeks of treatment with a NLRP3 inhibitor, MCC950 (10 mg/kg, i.p., 3 times per week), significantly limits hypertrophic remodeling and improves LV systolic and diastolic function (22). Consistent with a potential role of GPER in inflammasome deactivation, gene expression levels of key inflammatory genes, and cytokines related to inflammasome biology, including IL-18, IL-33, NLRP3, and caspase-1, were reduced in hearts of OVX-mRen2.Lewis rats treated with G1 compared with vehicle (unpublished data).

Whether G1/GPER-mediated anti-inflammatory responses are related to its effects on mast cells, as discussed previously, is not known. Mast cells are potent innate immune cells that accumulate in chronically inflamed tissues. The IL-1 family of cytokines, and particularly IL-33, activate mast cells and prime them to respond to inflammatory signals (160). If estrogen loss leads to a low-grade, chronic inflammatory state (161) in the female heart, the role of mast cells may evolve and continue to “feed the fire” via ongoing mediator release, such as chymase, thereby contributing to LV stiffness through hypertrophic cardiomyocyte and interstitial remodeling.

## GPER, LV Ejection, and Proximal Aortic Distensibility

LV ejection with respect to proximal aortic distensibility is another factor that contributes to diastolic function in the female heart. During systole, the long axis of the left ventricle normally shortens by pulling the aortic annulus toward the relatively fixed LV apex (162, 163). Displacement of the aortic annulus and sinotubular junction without concomitant movement of the aortic arch during systole promotes longitudinal stretch of the proximal aorta (162, 164, 165). While the aortic stretch that occurs during systole imposes a systolic load on the heart, it actually enhances early diastolic filling by serving as a reservoir for elastic energy (165). With loss of aortic distensibility due to advancing age, and presumably estrogen deficiency (163), or HFpEF (166) the displacement of the aortic annulus is reduced, as is the longitudinal long axis force or shortening of the left ventricle, leading to less stored elastic energy and impaired LV filling (167). Interestingly, postmenopausal women are more susceptible to the adverse effects of greater proximal aortic stiffness and pulsatile load on diastolic function and ventricular-arterial interaction than men of the same age (168–170). Moreover, the relationship between aortic impedance and diastolic dysfunction and ventricular-arterial coupling in women might be independent of LV remodeling (168), suggesting an additional contribution of aortic impedance to diastolic dysfunction in women.

Although the exact role of estrogen/GPER in aortic–ventricular interactions with respect to diastolic function is not known, recent preclinical studies suggest that GPER activation limits aortic stiffening and remodeling. GPER is expressed in both endothelial and smooth muscle cells of the aorta (59, 171) and GPER activation induces vasodilation similar to that seen with E_2_ (89, 172). In contrast to resistance arteries, GPER-induced vasorelaxation in the aorta is less robust (173) and the contribution of endothelial vs. smooth muscle signaling is more variable (59, 171). Interestingly, aortic GPER expression is downregulated in diabetes (174) but is functionally enhanced during pregnancy (171). In contrast to the extensive work assessing aortic reactivity, less is known about the impact of GPER on passive structural properties of conduit arteries. In the mRen2 rat model of hypertension, pharmacological activation of GPER in salt-loaded females significantly decreases aortic wall thickness without impacting blood pressure (175). GPER is also protective during carotid injury, where adenovirus-induced restoration of GPER protein expression is associated with a reduction in wall thickness in both male and female rats (176). While Ang II-induced hypertension is not impacted by GPER deletion, pulse pressure, and aortic wall thickness are significantly greater in female cardiomyocyte-specific GPER KO vs. wild-type mice (177). Therefore, while the role of GPER in proximal aortic distensibility has not yet been directly measured, published studies suggest that it most likely is another important factor impacting diastolic function.

## Translational Perspective

The sex-differential in the prevalence and incidence of human HFpEF is stark. Among women age >/ = 65 years, nearly 90% of new cases of heart failure are HFpEF. In the small number of men who develop HFpEF, the underlying characteristics differ markedly from that seen in women with predominantly ischemic heart disease, mildly dilated LV, and borderline/mild levels of systolic dysfunction. Thus, classic HFpEF is nearly exclusively a disorder of older, post-menopausal women. Despite this overwhelming magnitude of this profound biologic signal, its fundamental basis has not been systematically examined. Doing so could produce major insights into the initiation and progression of human HFpEF. Thus, the emerging data reviewed above has the potential to promote key advances in the understanding of human HFpEF and novel approaches to interrupting the pathways that lead to one of its precursors, diastolic dysfunction. This is greatly needed given the disappointing results of the recent PARAGON trial (178), and the other 7 large randomized trials of HFpEF that failed to achieve their pre-determined primary endpoint (179).

## Conclusion

Improvements in preventive medicine and health habits by positively lengthening the human life-span have brought to the forefront the impact of the menopause decline in women's cardioprotection. Estrogen-mediated cardiac health in women, using diastolic function as its monitor, is influenced by non-genomic mechanisms through GPER in the heart, in part by counteracting age and/or estrogen loss-dependent abnormalities in myocardial relaxation, cardiomyocyte Ca^2+^ homeostasis, mitochondrial function, and anti-hypertrophic/interstitial processes (Figure 2).

## Author Contributions

LG conceived and designed the manuscript. LG, Q-KT, and SL drafted the manuscript. Q-KT, HW, XS, LG, and SL created the tables and figures. DK provided the translational perspective. DK, CF, and CC were involved in critically reviewing and revising the manuscript for important intellectual content. All authors gave final approval of the manuscript to be published and agreed to be accountable for all aspects of the work.

### Conflict of Interest

The authors declare that the research was conducted in the absence of any commercial or financial relationships that could be construed as a potential conflict of interest.

## Abbreviations

ACE: angiotensin-converting enzyme
ANF: atrial natriuretic factor
Ang I: angiotensin I
Ang II: angiotensin II
Ang-(1-12): angiotensin-(1-12)
β-AR: beta-adrenergic receptor
BNP: brain natriuretic peptide
Ca^2+^: calcium
CaMKII: calcium/calmodulin-dependent protein kinase II
CICR: calcium-induced calcium release
E_2_: estradiol
EKG: electrocardiogram
eNOS: endothelial nitric oxide synthase
ER: Estrogen receptor
ERα: Estrogen receptor subtype α
ERβ: Estrogen receptor subtype β
ET-1: endothelin-1
FBS: fetal bovine serum
GPER: G-protein-coupled estrogen receptor
GPR30: G-protein-coupled receptor
HFpEF: heart failure with preserved ejection fraction
HTN: hypertension
I_Ca,L._: L-type calcium channels Ca_v_1.2
i.p.: intraperitoneal
IL: interleukin
ISO: isoproterenol
KO: knockout
LA: left atrium
LAP: left atrial pressure
LV: left ventricular
MCT: monocrotaline
MnSOD: manganese superoxide dismutase
mtDNA: mitochondrial DNA
NCX: sodium/calcium exchanger
NOX4: NADPH oxidases
OVX: ovariectomy
PAH: pulmonary arterial hypertension
PKA: protein kinase A
PLB: phospholamban
PMCA: plasma membrane calcium ATPase
pPLB: phosphorylated phospholamban
RAS: renin angiotensin system
ROS: reactive oxygen species
RV: right ventricular
RVT: right ventricular free wall thickness
RyR: ryanodine receptor
s.c.: subcutaneous
SD: Sprague Dawley
SLN: sarcolipin
SR: sarcoplasmic reticulum
SERCA2a: sarco/endoplasmic reticulum Ca^2+^ATPase 2a.
